# On-demand light-driven release of droplets stabilized *via* a photoresponsive fluorosurfactant

**DOI:** 10.1038/s41378-023-00567-3

**Published:** 2023-07-12

**Authors:** Guangyao Cheng, Qinru Xiao, Chit Yau Kuan, Yi-Ping Ho

**Affiliations:** 1 Department of Biomedical Engineering, The Chinese University of Hong Kong, Hong Kong SAR, China; 2 Centre for Novel Biomaterials, The Chinese University of Hong Kong, Hong Kong SAR, China; 3 Hong Kong Branch of CAS Center for Excellence in Animal Evolution and Genetics, The Chinese University of Hong Kong, Hong Kong SAR, 999077 China; 4 The Ministry of Education Key Laboratory of Regeneration Medicine, The Chinese University of Hong Kong, Hong Kong SAR, China

**Keywords:** Nanoscience and technology, Nanobiotechnology

## Abstract

Water-in-oil droplets have emerged as promising microreactors for high-throughput biochemical analysis due to their features of reduced sample consumption and automated operation. For a typical screening application, droplets are often trapped for continuous monitoring of the reaction over an extended period, followed by the selective retrieval of targeted droplets based on the after-effect of biochemical reactions. While techniques for droplet trapping are well developed, retrieval of targeted droplets mainly demands complicated device fabrication or sophisticated control. Herein, facile and rapid selective droplet release is achieved by utilizing a new class of photoresponsive fluorosurfactant based on plasmonic nanoparticles. The intense photothermal response provided by this novel photoresponsive fluorosurfactant is capable of vaporizing the fluorocarbon oil at the droplet interface under laser illumination, resulting in a bubble releasing a trapped droplet on demand. A fully automated fluorescence-activated droplet release platform has also been developed to demonstrate its potential for droplet-based large-scale screening applications.

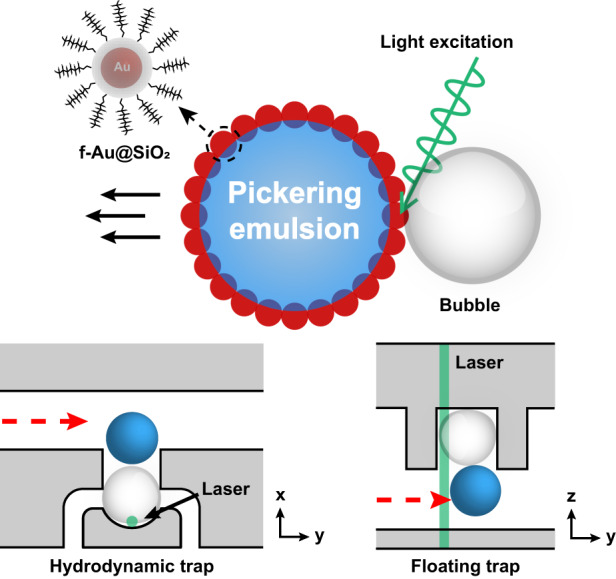

## Introduction

Droplet microfluidics has become a powerful technique for high-throughput analysis, as shown by many applications in drug screening, directed evolution, droplet digital PCR, and single-cell analysis^1–4^. Compared with conventional microwell plate-based assays, discretizing reagents to nanoliter or picoliter droplets benefits from reduced sample consumption, and thus, a reagent cost reduction by 3–6 orders of magnitude for an individual reaction can be achieved. Automated droplet manipulation (i.e*.*, merging, injection, and sorting) and detection have simulated and even surpassed labor-intensive sample manipulation in microwell plate-based assays^5,6^. However, for applications such as quantitative PCR and investigation of enzymatic reactions, droplets are often deposited in a trap for continuous monitoring of the reaction over an extended period. Accordingly, droplet traps have been previously developed and are broadly categorized as passive traps, such as those based on hydraulic resistance (termed “hydrodynamic traps” herein)^7^ and density differences (termed “floating traps” herein)^8^, and active traps, such as those utilizing electric fields^9^. Hydrodynamic and floating traps are widely used due to their simplicity and robustness by modulating the hydraulic pressure without additional force fields.

Additionally, the ability to selectively retrieve specific droplets is critical for downstream analysis, such as sequencing amplified DNA molecules post droplet PCR or the next-round evolution of enzymatic activity. Currently, trapped droplets can be released by three main strategies, including breaking the pressure balance^10–12^, regulating the pneumatic valve^13–15^, and light-induced bubbles^7,16,17^. When a droplet is held in a hydrodynamic trap, the droplet is balanced between the forward hydraulic pressure and the backward Laplace pressure. Therefore, droplets can be released either forwardly or backwardly by applying pressure to alter the pressure balance of trapped droplets. The increase in hydraulic pressure, or reduced Laplace pressure by lowering the interfacial tension, is able to release the droplet in a forward manner^10^. In contrast, to release the droplet against the main flow direction, a backward flow can be applied to reverse the hydraulic pressure, or an external force field, such as surface acoustic waves (SAW), can be used to generate extra pressure^11,12^. On the other hand, releasing droplets by regulating pneumatic valves typically requires a hydrodynamic trap where the droplets are trapped only when the valves are closed. Droplets held in such hydrodynamic traps are released when the corresponding pneumatic valve is open^13–15^. However, releasing droplets by breaking the pressure balance and regulating the pneumatic valves are constrained by complicated chip fabrication and sophisticated control since each trap requires an independent actuation source for selective release. Controlling the actuation to release droplets in different traps becomes progressively sophisticated when the trap number is scaled up. To this end, releasing droplets *via* light, more precisely by bubbles generated under laser illumination through the photothermal effect, has reduced the demand for sophisticated controllers^7,16,17^. Typically, the release is triggered by the excitation of a noncontacting laser beam that can be positioned among individual traps as intended. Therefore, the complexity of the controlled release does not expand as the trap number increases. Furthermore, the release *via* light-induced bubbles is applicable for both hydrodynamic and floating traps. However, in currently reported release methods based on light-induced bubbles, chip fabrication remains complicated due to the need to include a photothermal material, such as aluminum patches at each trap or a single photothermal layer; therefore, these methods are not widely used^7,17^. A 355 nm nanosecond pulsed laser is used to exclude the requirement of photothermal material and to simplify chip fabrication. However, 15 min is needed for each release event due to the low photothermal efficiency of pure water under UV light and the slow growth of bubbles^16^.

Currently, fluorocarbon oil is the most commonly used continuous phase in droplet microfluidics due to its gas permeability, chemical inertness, low toxicity to bioentities, and low solubility to polar molecules. Kinetic stabilization of water-in-fluorocarbon oil (W/O) droplets requires surfactants to be partially fluorophilic and hydrophilic, herein referred to as fluorosurfactants. For example, currently, available fluorosurfactants based on the short chain of 1H,1H,2H,2H-perfluoro-1-octanol (PFO)^18^, the block copolymer of perfluoropolyether-polyethylene glycol (PFPE-PEG), or the nonionic counterpart PFPE-tris(hydroxymethyl)methyl (PFPE-Tris)^19^ serve merely as a stabilizer at the droplet interface without providing additional functions. In regard to droplet manipulation, research mainly deals with designing and fabricating active components on chip, such as pneumatic valves and aluminum patches, as mentioned above. Previously, our group developed a photoresponsive fluorosurfactant based on fluorinated plasmonic nanoparticles (f-PNPs) capable of stabilizing water-in-fluorocarbon oil droplets efficiently. In addition, the fluorocarbon oil near the droplet interface could be superheated and vaporized at the millisecond time scale under 532 nm laser illumination due to the intense photothermal response of f-PNPs. Consequently, the generated vapor bubble was able to move the droplet along a designated direction^20^. In this study, we further applied the phenomena of light-driven droplet movement enabled by the photoresponsive fluorosurfactant to selectively release droplets in passive traps. Compared to previously reported strategies, especially those based on light-induced bubbles, our platform demonstrates great simplicity in chip fabrication and faster response.

## Methods

### Synthesis of fluorinated gold-silica core-shell nanoparticles (f-Au@SiO_2_)

f-Au@SiO_2_ was synthesized following a three-step procedure *via* wet chemistry as detailed and optimized in our previous publication^20^. Briefly, core gold nanoparticles (AuNPs, 13 nm) were synthesized through the reduction of HAuCl_4_ (254169-5 G, Sigma-Aldrich) by Na_3_Ct (W302600-1KG-K, Sigma‒Aldrich) in an aqueous solution. A silica shell (±5 nm) was then coated onto the AuNPs by the condensation of Na_2_SiO_3_ (338443-1 L, Sigma-Aldrich) with (3-aminopropyl)trimethoxysilane (APTMS) as the surface primer. Finally, the silica-coated AuNPs were functionalized with 1H, 1H, 2H, 2H-perfluorodecyltriethoxysilane (PFOTES) (667420-25 G, Sigma-Aldrich) to render the surface fluorophilicity. The peak absorption of the synthesized f-Au@SiO_2_ was empirically measured at 524 nm. The produced f-Au@SiO_2_ was weighed and redispersed in HFE-7500 (Novec^TM^, 3M^TM^) as a model among other commercially available fluorocarbon oils, such as FC-40 and FC-70 (Fluorinert^TM^, 3M^TM^), according to the designated weight percentage (1% w/w) for subsequent investigations.

### Construction of the fluorescence-activated droplet release (FADR) system

**Fig. 1:**
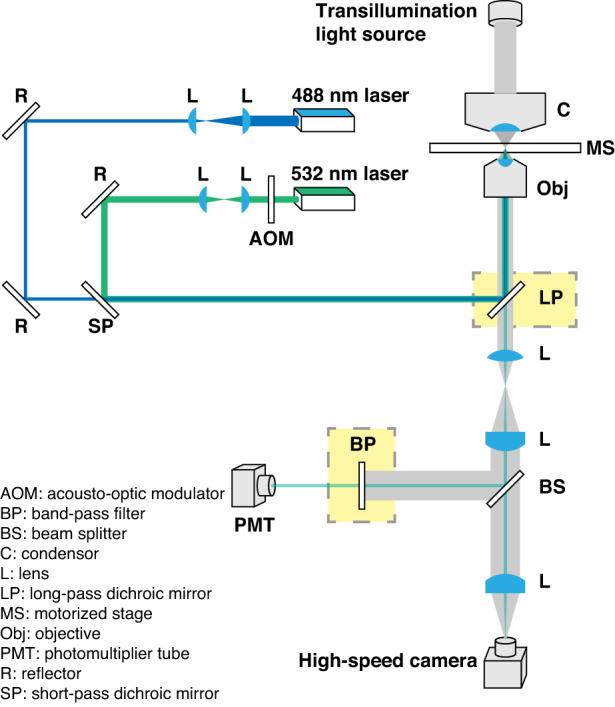
Configuration of the fluorescence-activated droplet release (FADR) system. The FADR system is customized to selectively release the droplets stabilized by f-Au@SiO_2_. A 488 nm laser is used to excite laser-induced fluorescence and the intended droplet is selectively released *via* the 532 nm laser

A conceptually novel fluorescence-activated droplet release (FADR) platform was constructed to allow the selective release of fluorescent droplets through a custom-built dual-laser system, as shown in Fig. 1. The release of droplets stabilized by f-Au@SiO_2_ was triggered by a 532 nm laser (power: 21.6 mW measured after the objective, beam waist: 6 μm), accompanied by an acousto-optic modulator (AOM) as an external shutter to control the on/off with a rising time of ~25 μs (Fig. S1, Supporting Information). The 532 nm laser used for droplet release was selected to maximize the photothermal conversion by f-Au@SiO_2_ with an extinction peak located at 524 nm^20^. A 488 nm laser (laser power: 2.4 mW, beam waist: 10 μm) was introduced as the excitation source for laser-induced fluorescence (LIF) occurring in each droplet. The emission was directed to a photomultiplier tube (PMT) and a high-speed camera through a beam splitter for quantification of the fluorescent signal and observation, respectively. A motorized stage was included to automatically move the microfluidic device, locating the laser beams at the intended position around a droplet for LIF detection or droplet release. The fluorescence intensity of each droplet was registered with the sampling rate of the analog-to-digital converter (ADC) set at 1 M/s. The detected signal was then stored upon the collection of all values. The intensity threshold was calculated based on the average fluorescence intensities from all droplets. The binarized intensity array was then generated to trigger the release. For a 5 × 5 array, the processing time was less than 1 ms.

### Design of the hydrodynamic trap and the floating trap

**Fig. 2:**
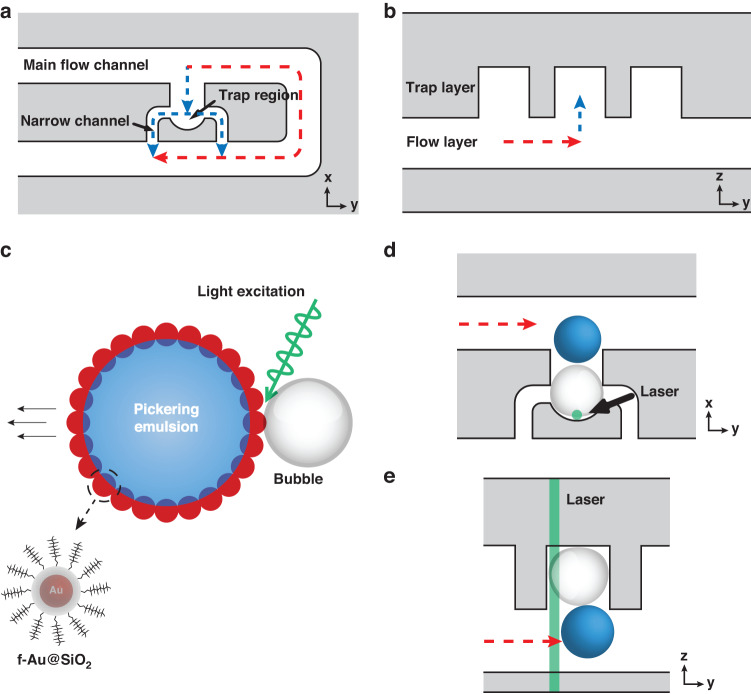
Schematic illustration of the trap-and-release process. **a** Hydrodynamic trap (top view). **b** Floating trap (side view). **c** Light-induced bubble generation for droplets stabilized by f-Au@SiO_2_. The photothermal response of f-Au@SiO_2_ vaporizes the oil phase under laser illumination. The laser propagates along the +z direction. The expansion of the vapor bubble transferring momentum to push the droplet in a designated direction is applied to release the droplet trapped in a **d** hydrodynamic trap and **e** floating trap

As shown in Fig. 2a, a hydrodynamic trap was configured by a main flow channel (red dashed line) and a trapping channel (blue dashed line) connected in parallel. The trapping channel consisted of a trap region and two narrow channels connected in series, whose hydraulic resistance was lower than that of the main flow channel. Therefore, the volumetric flow rate in the trapping channel was higher than that in the main flow channel. A droplet normally followed the flow entering the trap region, blocked by the narrow channel due to the reverse Laplace pressure. The trapping increased the hydraulic resistance in the trapping channel, thereby preventing following droplets from entering the filled trap. For Hagen–Poiseuille flow, the hydraulic resistance was calculated according to Eq. 1^7^:

1 $$R=\frac{C\left(\alpha \right)\mu L{\left(W+H\right)}^{2}}{8{W}^{3}{H}^{3}}$$

where *W* is the channel width, *H* is the channel height, *μ* is the fluid’s dynamic viscosity, *L* is the channel length, and *C(α)* is a constant dependent on the aspect ratio *α* (0 < *α* < 1) that can be approximated by a polynomial equation (Eq. 2):

2 $$C\left(\alpha \right)=96\left(1-1.3553\alpha +1.9467{\alpha }^{2}-1.7012{\alpha }^{3}+0.9564{\alpha }^{4}-0.2537{\alpha }^{5}\right)$$

Hence, to ensure effective trapping, a single-layer microfluidic device (constant channel height) containing a series of hydrodynamic traps was designed by engineering the geometry of the main flow channel and the trapping channel, according to Eq. 3:

3 $$\frac{{R}_{m}}{{R}_{t}}=\frac{{C}_{m}\left({\alpha }_{m}\right)}{{C}_{t}\left({\alpha }_{t}\right)}\frac{{L}_{m}}{{L}_{t}}{\left(\frac{{W}_{m}+H}{{W}_{t}+H}\right)}^{2}\frac{{W}_{t}^{3}}{{W}_{m}^{3}} > 1$$

where the subscripts *m* and *t* denote the main flow channel and trapping channel, respectively.

Compared with the traditional hydrodynamic trap, where only one narrow channel was positioned at the center behind the trap region, the hydrodynamic trap used in this study contained two narrow channels on both sides of the trap region, serving the following two purposes: (1) The laser was focused on the inner side of the trap region to generate a bubble for droplet release (Fig. 2d). With the narrow channel in close proximity, the bubble would escape through the narrow channel in the process of growth and fail the droplet release. (2) With two narrow channels connected in parallel, the hydraulic resistance was reduced by half. Therefore, a shorter main flow channel was needed, according to Eq. 3, rendering a reduced footprint of the chip. A 60-μm diameter hydrodynamic trap was used in this study for trapping a 50-μm diameter droplet, as optimized similarly in a previous study^7^. According to Eq. 3, the geometry of the main flow channel and narrow channels were optimized at *R*_*m*_/*R*_*t*_ = 2.5 with a channel height of 55 μm, where *R*_*m*_ and *R*_*t*_ were defined as the hydraulic resistance of the main flow channel and the trapping channel, respectively.

A double-layer floating trap was designed with a bottom flow layer and an upper trap layer with heights of 65 and 55 μm, respectively. A trap region of 60 μm × 60 μm was optimized for trapping a 50-μm diameter droplet, as shown in previous studies^8,17^.

### Fabrication of the microfluidic device

The two types of passive traps, namely, the hydrodynamic trap and floating trap, were fabricated by polydimethylsiloxane (PDMS) following the standard soft lithography technique. For the single-layer hydrodynamic traps, negative photoresist SU8-3050 (Kayaku Advanced Materials, Japan) was spun on a 4” silicon wafer at an intended speed for 30 s to obtain a specific height. The photoresist-coated wafer was exposed to UV (ABM, USA) through a film mask carrying the designed pattern (Microcad Photo-Mask Ltd, China). For the double-layer floating traps, spin coating and exposure were conducted twice, with the two film masks aligned *via* alignment marks. The handling of SU8, including the development of patterns and baking procedures, was performed following the manufacturer’s datasheet. Subsequently, PDMS prepolymer and curing agent (SYLGARD™ 184 Silicone Elastomer Kit, Dow Corning, USA) were thoroughly mixed in a 10:1 ratio and degased before pouring onto the SU8 master mold. The casted PDMS was cured at 70 °C for 2 hr, carefully peeled off, hole punched, and sealed with a cover glass by plasma treatment (Harrick Plasma, USA). The assembled devices were baked at 80 °C in an oven overnight to strengthen the bonding.

### Operation of the microfluidic device

W/O droplets (50 μm) stabilized by f-Au@SiO_2_ were generated by a flow-focusing droplet generator, as detailed in our previous study^20^. The as-produced droplets were then injected into the microfluidic traps at a volumetric flow rate of 0.5 μL/min for droplet trapping, followed by exchanging the continuous phase with pure HFE-7500 from a separate channel at a flow rate of 5 μL/min. Syringe pumps (Harvard Apparatus, USA) were used to control the volumetric flow rate, and PTFE tubing (Cole-Parmer, USA) was used as connections. The trapping efficiency was minimally interfered with by the flow rate (in a range of 0.5 to 10 μL/min) in the hydrodynamic traps, while the droplets were effectively trapped in the floating traps at a low flow rate (i.e*.*, <1 μL/min) due to the relatively slow floating upward of droplets to the trapping zone. Excess droplets were flushed away after trapping at a flow rate of 20 μL/min. Once the droplets were trapped, the HFE-7500 oil remained flowing at a flow rate of 1 μL/min during the release process.

## Results and discussion

As shown in Fig. 2a, a hydrodynamic trap functioned by directing a droplet to the trap region by optimizing the hydraulic resistance between the main flow channel (red dashed line) and the trapping channel (blue dashed line) connected in parallel^7^. On the other hand, floating traps were designed with a bottom flow layer and an upper trap layer, as shown in Fig. 2b. Due to the lower density of the aqueous droplet (water: 1 g/mL) than that of typical fluorocarbon oil (i.e*.*, HFE-7500: 1.614 g/mL), a flowing W/O droplet floated upward to the trap layer and ended up trapped in the trapping region^8,16,17^. Fluorinated gold-silica core-shell nanoparticle (f-Au@SiO_2_)-stabilized W/O droplets were trapped in the designed hydrodynamic and floating traps. As validated in our previous study^20^, f-Au@SiO_2_ at the droplet interface showed a photothermal response under 532 nm laser illumination, vaporizing the oil phase, as observed by a bubble generated at the droplet interface. The subsequent expansion of the vapor bubble transferred momentum to the adjacent droplet in a designated direction (Fig. 2c). This light-driven droplet movement enabled by the photoresponsive fluorosurfactant was capable of releasing the trapped droplets, as described in the following. In a hydrodynamic trap, the laser was focused on the droplet interface at the inner side of the trap region, generating a bubble to guide the droplet back into the main flow (Fig. 2d). In a floating trap, the laser was focused on the upper droplet interface, generating a bubble to push the trapped droplet downward to the flow layer (Fig. 2e).

**Fig. 3:**
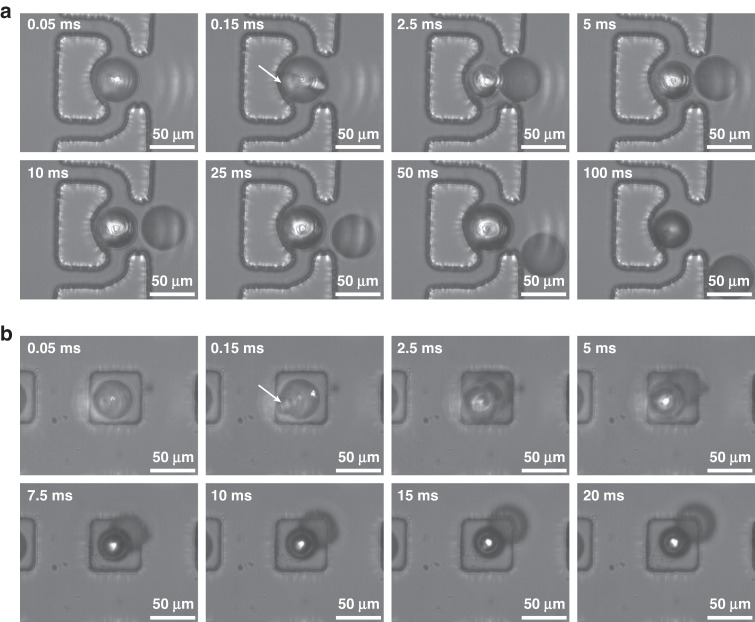
Release process of droplets stabilized by f-Au@SiO_2_ in passive traps. **a** For hydrodynamic trap, the 532 nm laser was focused on the droplet interface at the inner side of the trap. 50 ms of illumination generated a sufficiently sizable bubble to push the droplet back into the main flow for release. **b** For floating trap, the 532 nm laser was focused on the upper droplet interface. With a 5 ms of illumination, bubble generated on top of the droplet pushed the droplet downward to the flow layer for release

As a demonstration of trap-and-release in the hydrodynamic trap, droplets (50 μm) stabilized by f-Au@SiO_2_ were initially trapped in the hydrodynamic trap, as detailed in the Methods section. The injection of pure HFE-7500 ensured that the photothermal effect occurred solely at the droplet interface. With the decoration of f-Au@SiO_2_ at the droplet interface, these droplets remained stable against coalescence. The release of a trapped droplet by a light-induced bubble, as shown in Fig. 3a and Supplementary Video 1, was recorded *via* the high-speed camera. The 532 nm laser was focused on the droplet interface at the inner side of the trap region at *t* = 0 ms. Due to the intense photothermal response of f-Au@SiO_2_ at the droplet interface, the temperature near the laser illumination spot increased rapidly, vaporizing the oil phase to generate a vapor bubble at *t* = 0.15 ms (indicated by the white arrow in Fig. 3a). The vapor bubble continuously expanded in size until the laser was switched off (*t* = 50 ms). Given a fixed laser power (21.6 mW), the growth of the generated bubble was determined by the duration of illumination and observed to exhibit linear growth in droplet volume (Fig. S3, Supporting Information)^21^. In our experiments, the illumination was optimized at 50 ms, where the bubble was sufficiently sizeable to push the droplet toward the main channel and consequently released the droplet from the trap (Fig. S2a, Supporting Information). The released droplet then followed along the streamline of the main flow. The droplet release efficiency was reduced at higher oil flow rates due to the increased hydraulic pressure induced by the flow counteracting droplet release (Fig. S2b, Supporting Information).

For comparison, droplets (50 μm) stabilized by f-Au@SiO_2_ were used to validate the trap-and-release *via* the floating trap (trapping process detailed in the Methods section). A typical release process was shown in Fig. 3b and Supplementary Video 2. Briefly, the 532 nm laser was focused on the center of the upper droplet interface from *t* = 0 ms. At *t* = 0.15 ms, a vapor bubble was observed on top of the droplet (indicated by the white arrow). Due to the lateral confinement in the trap region, the expansion of the bubble pushed the droplet downward, rather than laterally, into the bottom flow layer and consequently released the droplet. Under the indicated settings, the illumination time was empirically optimized at 5 ms, where the release efficiency was higher than 95% (Fig. S4a, Supporting Information). The release efficiency was minimally altered by the flow rate in floating traps (Fig. S4b, Supporting Information). Note that the positioning of the laser beam was also crucial. As shown in Fig. 1, the laser propagated through the floating trap from the bottom. Therefore, positioning the longitudinal laser focus closer to the upper interface of the droplet ensured that the vapor bubble was generated on top of the droplet. In cases where the focus was not positioned properly, two vapor bubbles, one under and one above the droplet, were observed (Fig. S5, Supporting Information).

Compared with previously reported droplet release by light-induced bubbles^7,16,17^, the single release event was faster in our study, as demonstrated by releasing a droplet from the hydrodynamic trap and floating trap (i.e., 50 and 20 ms, respectively). Furthermore, the vapor bubble generation required relatively lower laser power than the existing strategies due to the intense photothermal response produced by f-Au@SiO_2_ at the droplet interface. The traps used in our study were fabricated *via* standard soft lithography with PDMS; however, the light-driven release was readily applicable to traps produced by other transparent materials. Additionally, our demonstrated device and trap-and-release process were biocompatible, as verified by the minimally altered viability of human embryonic kidney cells (HEK 293), as a model cell line, under the illumination of the employed settings (Figs. S6, S7, Supporting Information). As a comparison of the two traps, the footprint of the floating trap (60 μm × 60 μm) was much smaller than that of the hydrodynamic trap (600 μm × 225 μm), whose fabrication processes was simpler. Moreover, releasing a droplet from the floating trap required shorter laser illumination (5 ms) compared to that for the hydrodynamic trap (50 ms). In hydrodynamic traps, the hydraulic pressure induced by the constant flow counteracted the droplet release, resulting in reduced release efficiency under a higher oil flow rate, and thereby a longer illumination was required to produce a sizeable vapor bubble for release (Fig. S2, Supporting Information). In contrast, based on Bernoulli’s principle, the constant flow in floating traps led to lower pressure in the flow layer, easing the droplet release with a smaller bubble. As shown in Fig. S4 (Supporting Information), droplets were released effectively across a range of flow rates in floating traps. Finally, the operation of floating traps was considered practically manageable with less probability of channel blocking. Herein, considering the ease of operation and device footprint, floating traps were used for the subsequent demonstration of our proposed fluorescence-activated droplet release.

**Fig. 4:**
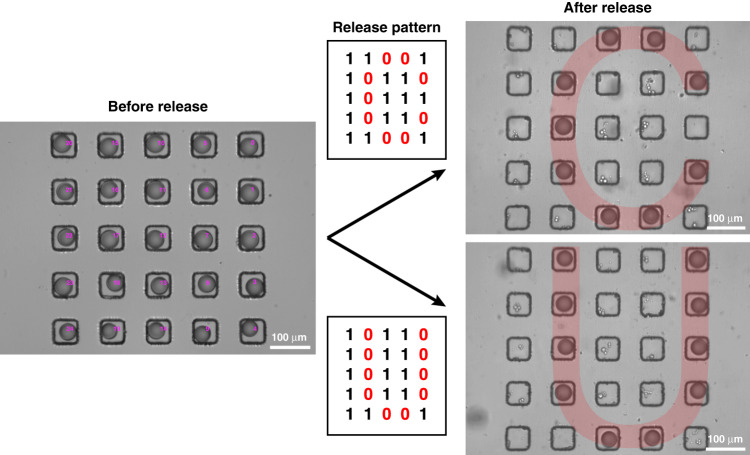
Selective release of droplets stabilized by f-Au@SiO_2_ in a 5 × 5 floating trap array. The release pattern was defined by a 5 × 5 binary number array. The letters “C” and “U” were formed by droplets trapped in the 5 × 5 floating trap array after automated and selective release

Droplets stabilized by f-Au@SiO_2_ were trapped in a 5 × 5 array of floating traps, as shown in Fig. 4. Note that the release was demonstrated within the 5 × 5 array to prevent drifting of the longitudinal focus during scanning. The geometry of each floating trap was identical to the one mentioned above, whereas the gap between two adjacent traps was set at 100 μm. Customized LabVIEW and Python programs were used to automate the process of selective release of trapped droplets. The floating trap array was filled with droplets, followed by detection with a You Only Look Once (YOLO) model trained by a custom dataset^22^. The coordinates of droplets were then calculated with reference to the laser position. A binary number array with the same shape as the floating trap array (5×5 herein) was used as an input to define the release pattern, where “1” and “0” referred to an event of released and retained droplets, respectively. With a defined release pattern (letter “C” or “U” in the example of Fig. 4), the droplets to be released were indexed, and a trajectory map was generated for the movement of the motorized stage, which then moved the to-be-released droplets toward the laser spot. Droplets located closer to the upstream of flow were released first, followed by those located downstream to prevent the released droplets from re-entering empty traps farther downstream. Laser illumination for each release event was typically set at 5 ms, while a 0.05 ms laser pulse produced a satisfactory success rate as well (Supplementary Video 3). The generated vapor bubbles were either pushed out by the oil flow at a flow rate of 20 μL/min or dissipated after the laser switched off without interfering with subsequent release events. With this trap-and-release system, the automated and selective release of droplets trapped in the 5 × 5 floating trap array, forming the letter “C” or “U,” shown in Fig. 4, was accomplished within seconds.

**Fig. 5:**
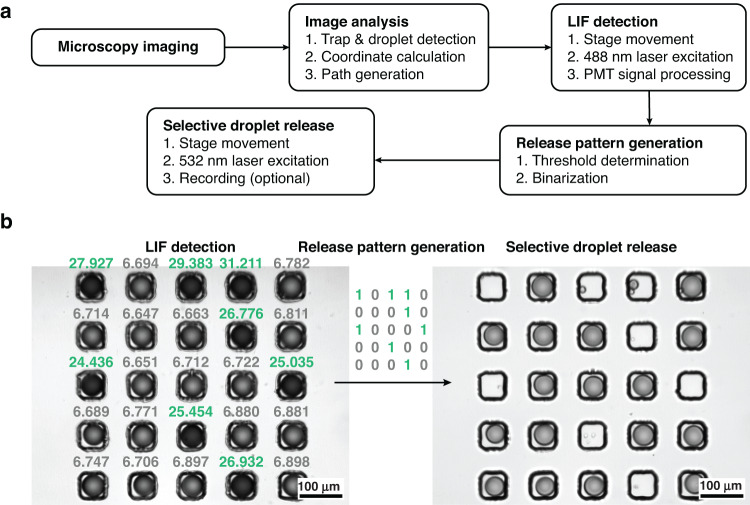
Demonstration of the fluorescence-activated droplet release (FADR) platform. **a** Workflow. **b** Selective release of fluorescently positive droplets that are dark in appearance under the bright field and have higher LIF intensity in a 5 × 5 floating trap array

In addition, FADR was demonstrated following the workflow, as shown in Fig. 5a. Fluorescently positive and fluorescently negative droplets were separately generated and stabilized by f-Au@SiO_2_. The two types of droplets were then mixed and randomly trapped in a 5 × 5 floating trap array, as shown in Fig. 5b (left). The dispersed phase for the fluorescently positive droplets consisted of fluorescein isothiocyanate (FITC, 0.01 mg/mL) and trypan blue (0.2%), enabling the droplets to show green fluorescence under blue light excitation and become dark under bright-field imaging. On the other hand, fluorescently negative droplets contained merely DI water. The floating trap array filled with droplets was imaged, and the droplets were coordinated *via* the abovementioned methodology. Subsequently, the longpass dichroic mirror (DM) was switched to allow the 488 nm laser to illuminate the droplets for LIF excitation. In this case, all droplets were moved underneath the laser focus sequentially to acquire LIF intensities detected by the PMT. As shown in Fig. 5b (left), the detected LIF intensity was collected and labeled for each droplet. The dark droplets showed higher LIF intensity (green values) than the bright droplets (gray values), consistent with the anticipated droplet properties for the two groups. A threshold of LIF intensity was then determined for the generation of the release pattern, where the LIF intensity above and below the threshold (i.e., 13 in this specific experiment determined by the average fluorescence intensity of all droplets) was binarized to 1 and 0, respectively. A binary number array with the same shape as the floating trap array (5 × 5 herein) was generated to guide the selective droplet release. The longpass DM was then switched to allow the passing of the 532 nm laser for droplet release. With the defined release pattern from LIF intensity, fluorescently positive droplets were released one by one *via* the same procedures described above. Fluorescently negative droplets were unaltered in the floating trap array, as shown in Fig. 5b (right). Note that droplet splitting was observed occasionally during bubble generation, consequently leaving a tiny drop in the trap, as indicated in Fig. 5b (right). On the other hand, the selective release of fluorescently negative droplets can be achieved by simply reversing the polarity of the binary number array generated from the detected LIF intensity. The fully automated workflow controlled by a custom LabVIEW and Python program completed the FADR within seconds. After selective release and retrieval downstream, all remaining droplets were released and washed out for another round of the trap-and-release process. Therefore, the trap was reusable without any functional damage.

## Conclusions

In summary, the novel photoresponsive fluorosurfactant based on f-PNPs has enabled the selective release of droplets trapped in hydrodynamic traps and floating traps. The intense photothermal response of f-PNPs at the droplet interface enables the vaporization of the oil phase under laser illumination, generating a bubble to displace the trapped droplets for selective release. The release event can also be triggered *via* fluorescence, the most commonly used staining in biochemical reactions. Among the reported release methods *via* light-induced bubbles, our release platform has the salient features of simple chip fabrication, low laser power, short response time, facile scale-up ability, and reusability of microfluidic traps. Furthermore, the heat is presumably confined locally around the laser spot since the bubble is generated within submilliseconds. The laser-induced damage against the biomolecules in the droplet is expected to be minimal; however, further confirmation is needed with an array of biomolecules involved in the targeting biochemical applications. Based on these results, our automated trap-and-release platform enabled by f-PNPs is anticipated to facilitate droplet-based large-scale screening applications, where fast and precise retrieval of targeting droplets after extended incubation and observation are in critical need.

## Supplementary information

Supporting Information

Supplementary Video 1

Supplementary Video 2

Supplementary Video 3
