# Supplementary material for: On-demand light-driven release of droplets stabilized via a photoresponsive fluorosurfactant
Source: Microsyst Nanoeng. 2023 Jul 12;9:89. doi: 10.1038/s41378-023-00567-3 (PMC10336138; doi:10.1038/s41378-023-00567-3)
Supplement: Supplementary file 1 — Supporting Information [file 41378_2023_567_MOESM1_ESM.docx]

Supporting Information

On-Demand Light-Driven Release of Droplets Stabilized by Photo-Responsive Fluorosurfactant

*Guangyao Cheng,^1^ Qinru Xiao,^1^ Chit Yau Kuan,^1^ and Yi-Ping Ho^1,2,3,4*^*

^1^ Department of Biomedical Engineering, The Chinese University of Hong Kong, Hong Kong SAR, China.

^2^ Centre for Novel Biomaterials, The Chinese University of Hong Kong, Hong Kong SAR, China.

^3^ Hong Kong Branch of CAS Center for Excellence in Animal Evolution and Genetics, The Chinese University of Hong Kong, Hong Kong SAR, China.

^4^ The Ministry of Education Key Laboratory of Regeneration Medicine, The Chinese University of Hong Kong, Hong Kong SAR, China.

Email: [ypho@cuhk.edu.hk](mailto:ypho@cuhk.edu.hk)

**Cell Viability**

*Live/dead fluorescence staining.* Human embryonic kidney cells (HEK293, ATCC catalog number CRL-1573) were trypsinized and incubated with the 1× stain solution of LIVE/DEAD™ viability/cytotoxicity kit (TheromoFisher, USA) at 37°C under 5% CO_2_ for 15 minutes. The mixture was then introduced into the droplet microfluidic following the procedures described in the Method Section, allowing the stained cells being encapsulated into f-Au@SiO_2_ stabilized droplets. The droplets containing cells before and after the laser illumination were observed under an inverted confocal laser‐scanning microscope (C2 Plus, Nikon, Japan). Bright-field and fluorescent images were taken using a 60× objective (Nikon, 60×/NA1.4, oil immersion, Japan). The green fluorescent signals from Calcein-AM, as an indicator of live cells, were acquired by the excitation of 488 nm laser, and the emission was filtered through the filter of 525/50 nm (bandpass).

*Trypan blue exclusion*.^1^ Cells were stained with 0.4% trypan blue solution (Thermo Fisher, USA) and observed under an inverted phase contrast microscope (ECLIPSE Ti, Nikon, Japan), where nuclei of viable cells appeared phase bright and nuclei of dead cells appeared blue.

**Supplementary figures:**

**
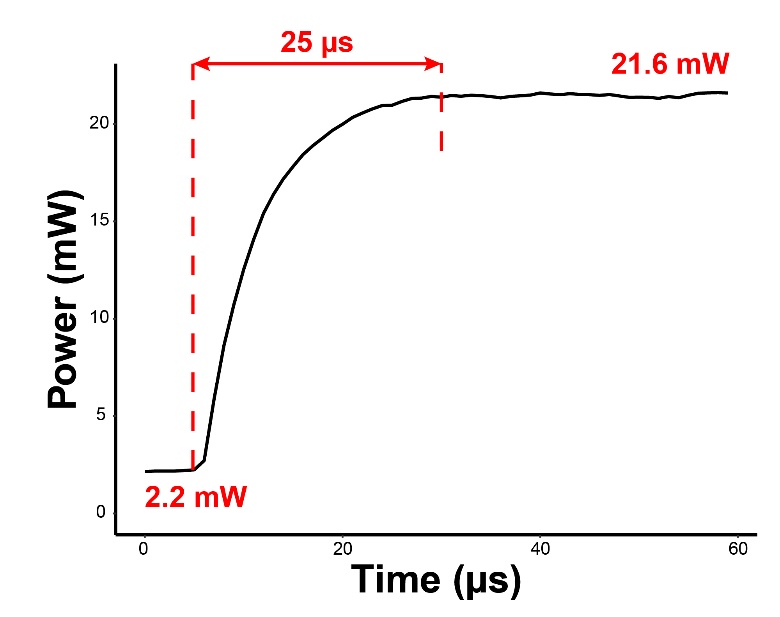
**

**Figure S1. Laser (532 nm) power as a function of time. The AOM shutter was fully opened at 25 μs.**


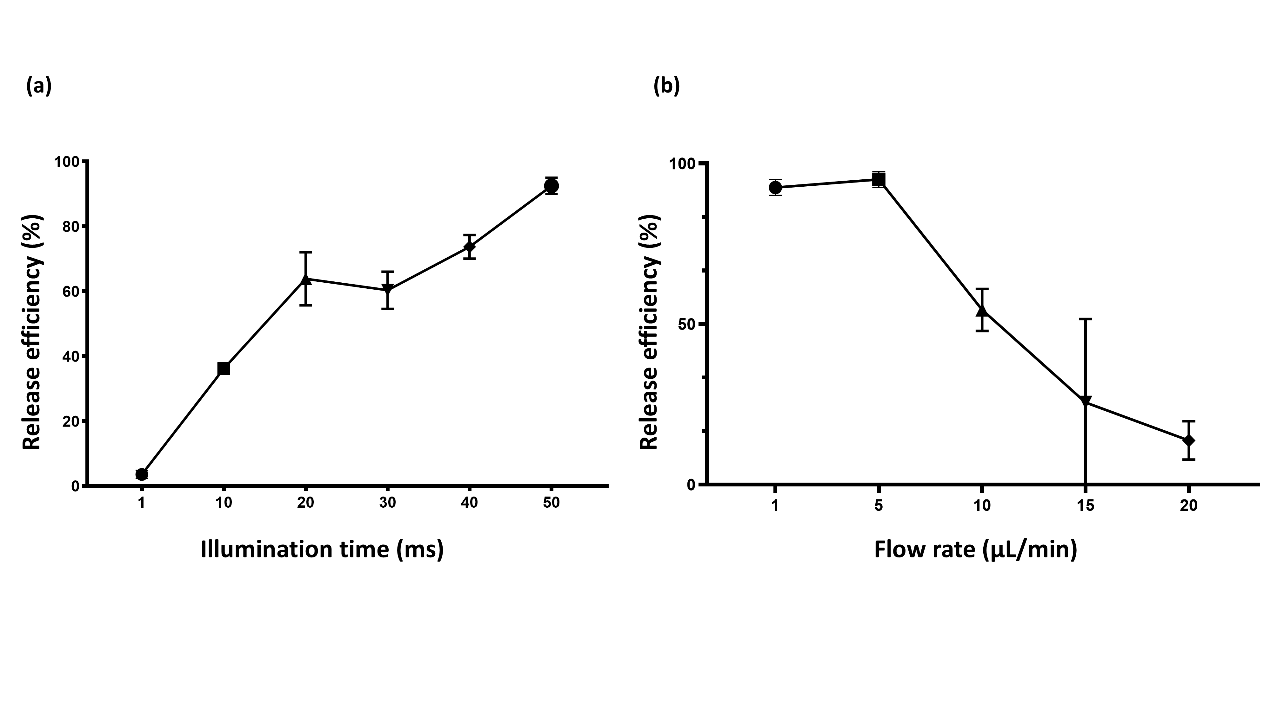


**Figure S2. Characterization of droplet release efficiency in hydrodynamic traps.** (a) The release efficiency was followed within a range of laser illumination time. The illumination time was empirically determined at 50 ms, showing a release efficiency > 95%. (b) The release efficiency was quantitatively followed within a range of flow rates.

**
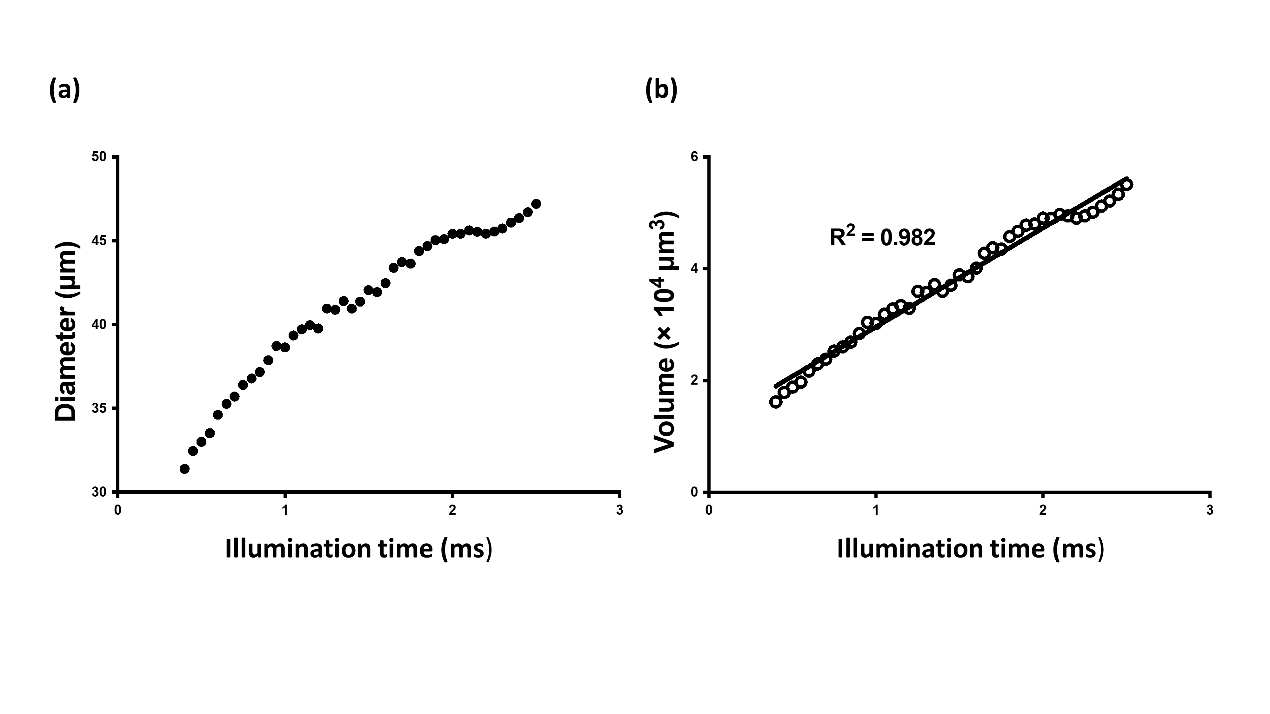
**

**Figure S3. The growth of bubbles under the illumination of 532nm (21.6 mW) was plotted as a function of the illumination time versus (a) the diameter of a bubble, and (b) the volume of a bubble.** A linear relationship between the illumination time and the volume of a bubble was observed, similar with a previous study.^2^


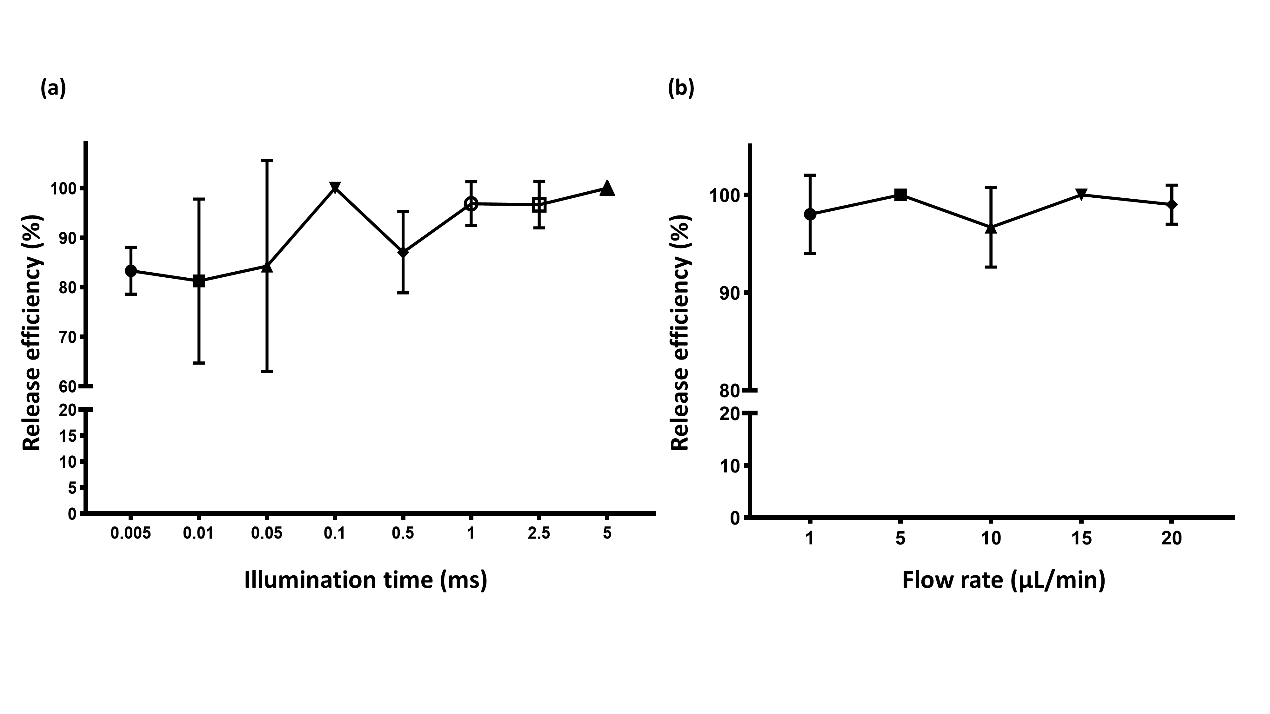


**Figure S4. Characterization of droplet release efficiency in floating traps.** (a) The release efficiency was followed within a range of laser illumination time. The illumination time was empirically determined at 5 ms, under which the release efficiency was observed more than 95%. (b) The release efficiency was quantitatively followed within a range of flow rates.

**
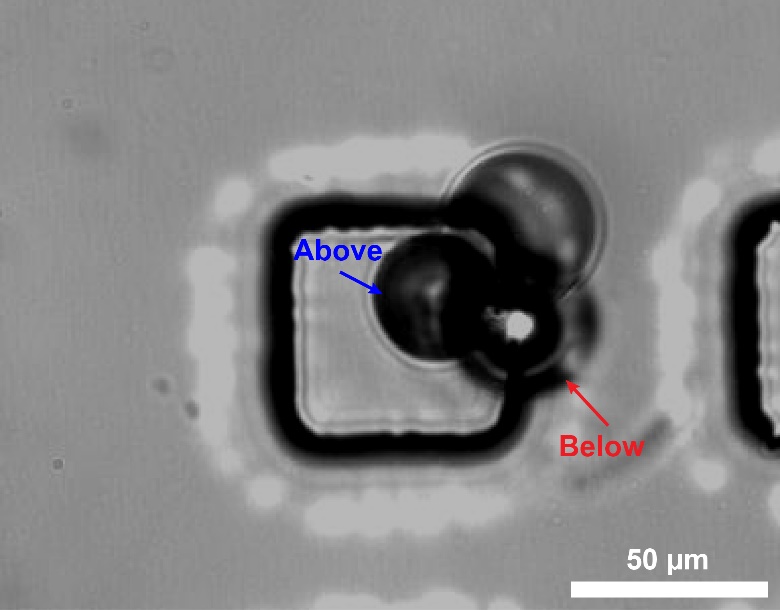
**

**Figure S5. Illumination at non-optimal longitudinal focus, two vapor bubbles were observed under and above the droplet.**

**
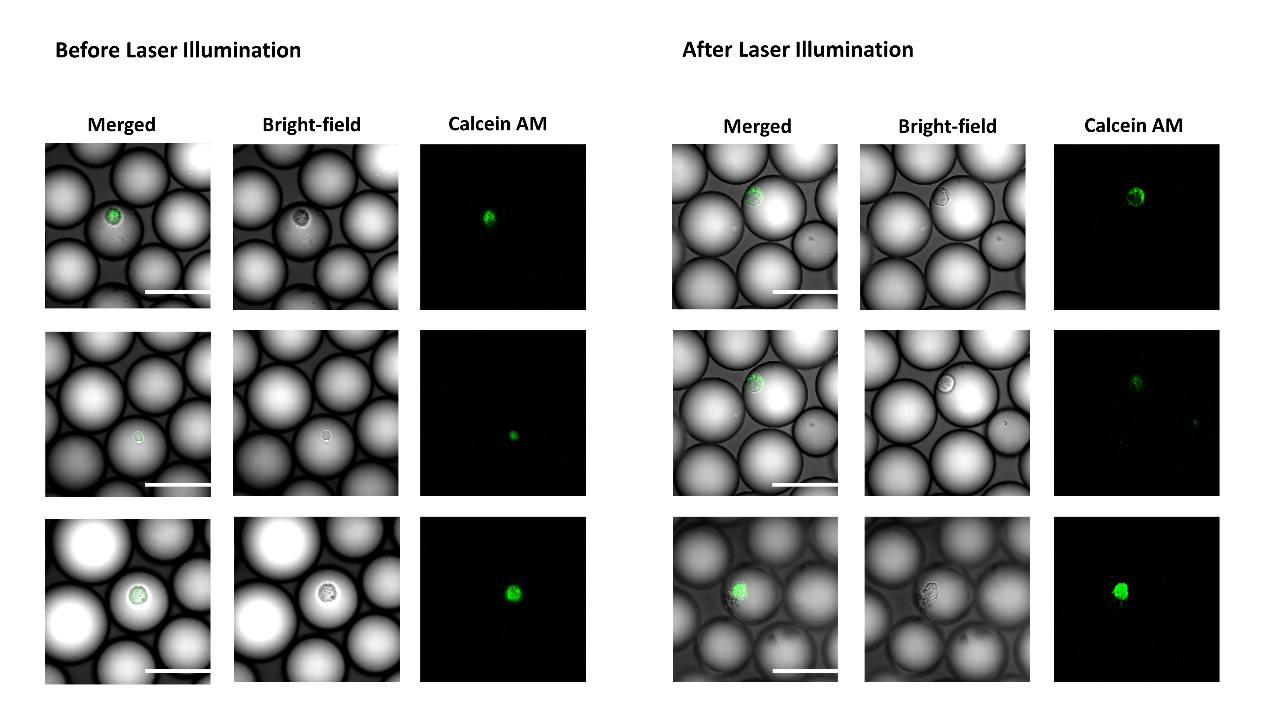
**

**Figure S6. Representative images showing cell viability before/after the laser illumination of employed settings.** Bright-field and fluorescent images were taken from droplets containing at least one HEK 293 cell. Live cells were stained by Calcein-AM, showing green fluorescence. Scale bar 50 μm.


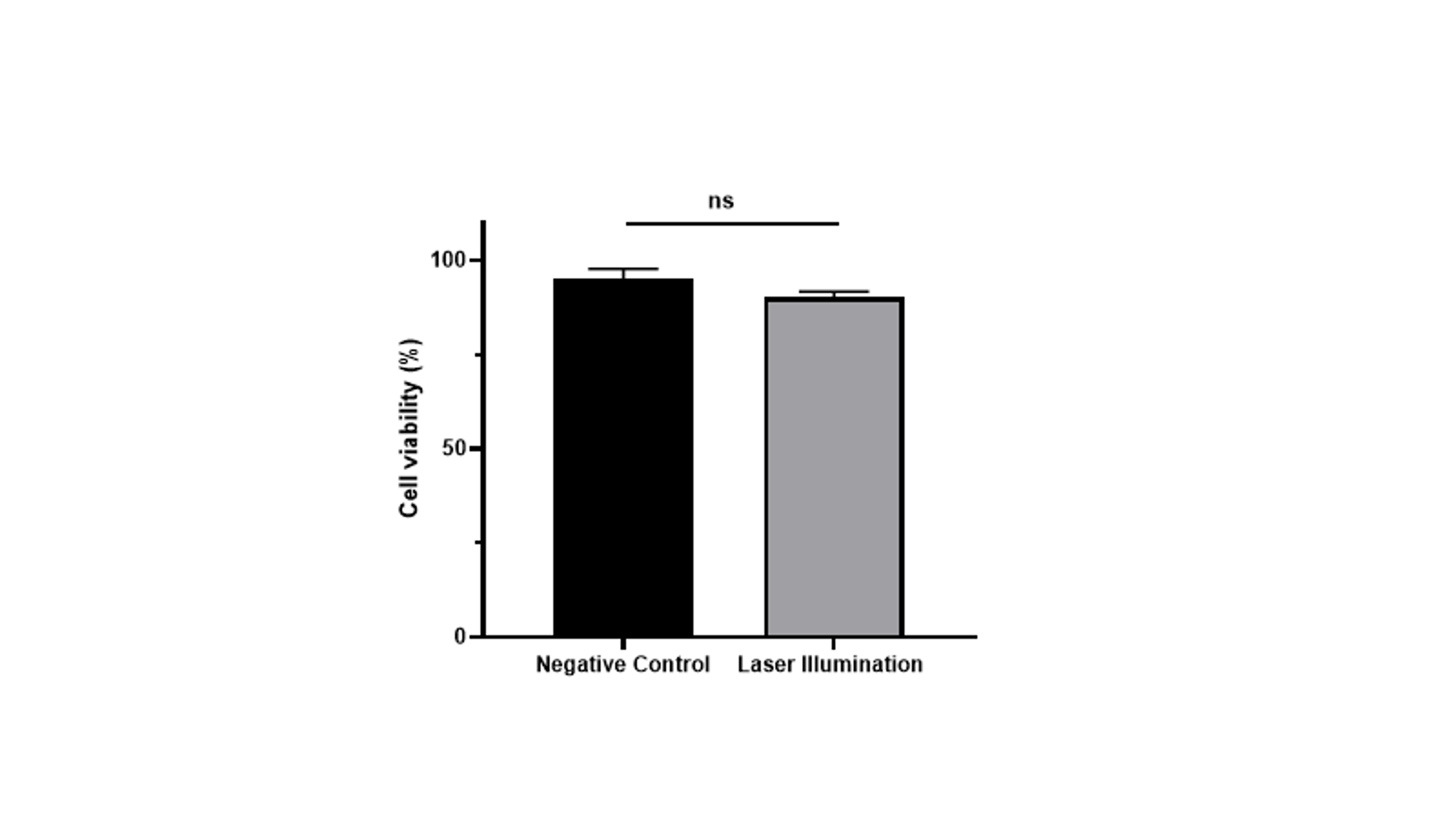


**Figure S7. Cell viability quantified by trypan blue exclusion.** The cell viability was calculated by the ratio of living cells versus total cells. At least 200 cells were analyzed for each experiment. Statistical significance was evaluated by two-tailed unpaired Student’s *t*-tests, *ns*: not significant.

**References:**

(1) Strober, W. Trypan Blue Exclusion Test of Cell Viability. *Current Protocols in Immunology* **2015**, *111* (1), A3.B.1-A3.B.3. https://doi.org/10.1002/0471142735.ima03bs111.

(2) Zaytsev, M. E.; Lajoinie, G.; Wang, Y.; Lohse, D.; Zandvliet, H. J. W.; Zhang, X. Plasmonic Bubbles in *n* -Alkanes. *J. Phys. Chem. C* **2018**, *122* (49), 28375–28381. https://doi.org/10.1021/acs.jpcc.8b09617.
